# Cause of neonatal deaths in Northern Ethiopia: a prospective cohort study

**DOI:** 10.1186/s12889-016-3979-8

**Published:** 2017-01-11

**Authors:** Hayelom Gebrekirstos Mengesha, Berhe W. Sahle

**Affiliations:** 1 College of Medicine and Health Science, Adigrat University, Adigrat, Ethiopia; 2 School of Pharmacy, Mekelle University, Mekelle, Ethiopia; 3 School of Public health, Mekelle University, Mekelle, Ethiopia

**Keywords:** Cause of death, Neonatal mortality, Ethiopia

## Abstract

**Background:**

Despite the significant reduction in childhood mortality, neonatal mortality has shown little or no concomitant decline worldwide. The dilemma arises in that the lack of documentation of cause of death in developing countries, where registration of vital events is virtually nonexistent. Understanding of the causes of death in neonates is important to guide public health interventions. The present study identifies the common causes of neonatal death in Ethiopia.

**Methods:**

A prospective cohort study was conducted among neonates born between April 2014 and July 2014 in seven hospitals, in Tigray region, Ethiopia. Mothers were interviewed by midwifes respecting risk factors and infant survival. For neonates who died in hospital, causes of death were extracted from medical records, whereas a verbal autopsy method provided presumptive assignment of cause of death for those infants who died at home.

**Results:**

Of the1152 live births, there were 68 deaths (63 per 1000 live births). Two thirds of deaths were attributable to prematurity 23 (34%) or asphyxia 21 (31%). Slight variance was seen between the morality patterns in early and late neonatal periods. In the early neonatal period, 37% were due to prematurity, while asphyxia (35%) was more common in the late neonatal period. All infection-related deaths occurred in neonate-mother dyads from rural areas.

**Conclusion:**

Prematurity, asphyxia, and infections were the leading causes of neonatal deaths in Tigray region during the study period. Causes of deaths identified during early and late neonatal mortality differed, which clearly indicates the need for responsive and evidence-based interventions and policies.

## Background

Globally, the United Nations Inter-agency Group for Child Mortality Estimation of Child Mortality reported 2.8 million neonatal deaths in 2013 [1]. Neonatal deaths account for about 44% of under-five (U5) deaths worldwide and 62% of infant deaths in Ethiopia [1, 2]. Neonatal mortality rate (NMR) has decreased globally since 1990, although the rate of decline is lower compared to theU5s cohort [1, 3].

NMR is unacceptably high, especially in Sub-Saharan Africa which exhibits the least progress and carries the highest burden of neonatal deaths worldwide [1, 2]. The regional NMR is 31 per 1000 live births, as compared to the global NMR of 20 per 1000 live births [1]. Globally, preterm birth complications (35%), intra-partum complications (mainly intrapartum hypoxia) (23%), and sepsis including meningitis (15%) account for three quarters of all neonatal deaths [1]. Evidence shows that up to 50% of neonatal deaths can be prevented through cost-effective interventions that may apply before, during, and after delivery. A large multi-national study found that preterm and intrapartum complications are the major cause of early neonatal death, while sepsis was the major cause of death for late neonatal mortality [4]. In the same study, preterm birth was the leading cause of neonatal mortality in all 194 countries [4].

In the last two decades, the Government of Ethiopia has implemented the health sector development program to improve access and quality of health services to all segments of the population [5]. The healthcare reforms focus on ensuing access and utilization of primary healthcare services including to those in remote and rural areas [5]. Skilled delivery, and antenatal care has increased from 10 and 34% in 2011 to 16 and 41% in 2014 respectively [6, 7]. Institutional delivery increased from 5% in 2000 to 16% in 2014 in Ethiopia overall, but, in Tigray, the rate is 27% [7]. Further, postnatal care within 48 h of delivery increased from 2% in 2000 to 13% in 2014 [7]. Despite these positive indicators, neonatal mortality remains unacceptably high in Ethiopia. According to the 2011 Ethiopian Demographic and Health Survey (EDHS), the NMR was estimated to be 37 per 1000 live births [6]. It only declined by 1 per 1000 live births from the 2005 EDHS estimates [8]. Evidence also showed significant disparity in rates across various administrative regions ranging from 21 to 62 per1000 live births [6].

In developing countries deaths are often not registered and causes of death are not assigned to 99% of deaths. Thus, few studies are available, which lack generalizability because of their inadequate representation [9–11]. Like variable neonatal mortality prevalence, the causes of neonatal death vary within and between countries due to variability in distribution of risk factors and access to health care [1, 12]. As a result local programming and decision making must be built around local causes of death to be meaningful [13]. Separate causes of death estimates are also required for the early and late neonatal periods due to variation in the cause of death patterns between these periods [14–17].

Findings from previous studies in Ethiopia revealed that most neonatal deaths were due to sepsis, asphyxia, birth injury, tetanus, preterm birth, and congenital malformations [9, 10]. Limitations in such studies included narrow geographic inclusion [9, 10], and methodological approaches including population based verbal autopsy [9, 11] and retrospective household surveys [6]. Therefore, the need for a large prospective cohort study in Ethiopia was made obvious into the causes of early and late neonatal deaths.

## Method

### Study design and setting

A prospective cohort study including live births between 1 April 2014 and 31 July 2014 was conducted in seven randomly selected hospitals in Tigray Region of Ethiopia.

The Ethiopian health care has a decentralized four-tier system consisting of primary health care, district, zonal, and specialized hospitals. The primary health care includes rural health posts, nested into health centers serving 25,000 populations. District hospitals are expected to serve catchment populations of 250,000. Zonal and specialized hospitals serve 500,000 and five million people, respectively. There are a total of 15 public hospitals: six zonal, one specialized and eight district hospitals in Tigray region. Comprehensive maternal and newborn services, including cesarean section and delivery services, are provided free of charge.

In the present study, seven hospitals were randomly selected from the 15 hospitals using a lottery method [18, 19].

All mothers who had a live birth in the study hospitals during the study period were eligible for inclusion. Exclusion criteria included those arriving at the hospitals after six hour of delivery and/or had a psychiatric illness.

### Sample size

This is a sub-study undertaken to estimate NMR in the region. The study included 1162 mother-neonate dyads. Details of sample size calculation and sampling procedure have been published previously [20].

### Data collection and follow up

Data related to neonates, mothers, and pregnancies were collected using a pretested structured questionnaire. The International Classification Disease Version 10 (ICD-10) verbal autopsy questionnaire was adapted to collect verbal autopsy data [21].

Data were collected by midwifes working in the respective hospitals. The survival status of neonates after discharge was ascertained by making follow-up phone calls every week to the end of the neonatal period. Those who could not be reached by phone were visited by health extension workers (i.e., primary health workers who live in the community) every week to ascertain the status of the neonate who then notify the outcome to the study midwife. Clinical characteristics related to the mother and her baby were either extracted from medical record or collected at baseline. Details of follow up have been published previously [20].

### Assigning the probable cause of death

For neonates who died at home after discharge, probable causes of death were assigned by physicians using the ICD-10 coding [21]. For neonates who died in the health facility, cause of death was assigned by physicians, who work in the study hospitals, based on national guidelines [22] and the Wigglesworth classification with the revised decision tree modified from the neonate and stillbirth single cause of death classification [23, 24] and a criteria derived and slightly modified from elsewhere [25] to assign single cause of death for all neonates. When death occurred at home, data related to the illness were recorded using the standard WHO verbal autopsy questionnaire and supplemented with clinical hospital records from delivery and discharge to provide additional information on the cause of death. Two physicians independently reviewed the completed verbal autopsy questionnaires to assign underlying cause of death using the ICD-10coding [21]. Physicians assigned up to three underlying causes using the three digit ICD-10 coding. Consensus was sought to establish a cause of death. When disagreement arose, the verbal autopsy form was sent to a third independent physician and cause of death was reported if this physician agreed with either of the two physicians. In instances when no agreement was reached, the principal cause of death was reported as ‘unknown’.

### Training of verbal autopsy reviewers and data collectors

Data collectors received one day training on data collection, on how to deal with the hospital-based clinical diagnostic data, the verbal autopsy form, and the recruitment and follow- up of mothers with their neonates. Physicians were also trained for one day on how to review the questionnaire in order to assign ICD-10 code for cause of neonatal death.

### Measurement

Birth weight was measured using standard weight measuring balances used in the study hospitals. Low birth weight was defined as a birth weight of <2500 g, and normal 2500 g to 4000 g, and macrosomia ≥ 4000 g. Gestational age was determined based on the mother’s last menstrual period. Probable causes of death were defined as: 1) prematurity if the neonate was born before 37 completed weeks, had surfactant deficiency, intraventricular hemorrhage, respiratory distress syndrome, and/or necrotizing enterocolitis; 2) asphyxia included a failure to initiate spontaneous respirations and/or Apgar score of less than 3 in the first 5 min from delivery, and neonatal hypoxic/ischemic encephalopathy yielding an Apgar score at 5 min of less than 7;3) infection included any possible source, such as sepsis, pneumonia, meningitis, and/or respiratory tract infections; and 4) congenital abnormality included a potentially lethal congenital malformation (e.g., congenital cardiac defect, spina bifida, congenital syndromes, gastro-intestinal malformation). Further, “unknown” cases included deaths which did not have a specific cause, have little information, or no determined cause based on the ICD-10 and hospital-based clinical diagnosis.

In this study, early neonatal mortality was defined as the probability of death before 7 completed days of life and late neonatal mortality defined as the probability of dying between 7 completed days and before 28 completed days. Similarly, the cause specific mortality rate was defined as death of a neonate during the first 28 days of life attributed to specific cause per 1000 live births.

Reported income was categorized into low (<500 Ethiopian birr (ETB)), medium (500–1500 ETB) and rich (>1500 ETB). Residence was classified as ‘urban’ and ‘rural’. Urban is defined as a locality with 2000 or more whose inhabitants are primarily engaged in non-agricultural activities. All participants not meeting the urban definition were considered ‘rural’.

Educational status was first categorized as ‘unable to read and write’, ‘able to read and write but no formal education’, ‘grade 1–4’, ‘grade 5–8’, ‘grade 9–12’, ‘college and above’. During analysis, we merged the select categories due to small sample size. Specifically, our final categories were ‘unable to write & read or no formal education’, ‘primary education (grades 1–8)’, ‘secondary education (grades 9–12)’ and ‘tertiary education (college and above)’.

Intermediate factors included maternal, neonatal, and health service related considerations. These were operationalized as gender of the neonate categorized as female/male; distance from respondents’ home to the health facility (measured or calculated in walking time converted to kilometers(km) reported as <5 km, 5–10 km and > =10 km distance).

### Data management and analysis

Descriptive statistics included mean, standard deviation, frequency and associated percentages where appropriate. Associations between sociodemographic factors and cause of death were examined using chi-square. We classified each neonatal death in the hierarchical order of prematurity, asphyxia related, infection, congenital abnormalities, and other (unknown) cause of deaths. Data cleaning and analysis was done using Stata™ Version 12.

## Results

### Characteristics of mothers and neonates

Responses were obtained from 1162 mother-neonate dyads (see Table 1 for demographic details). Ten observations were excluded from the analysis due to incompleteness of the data. The mean (±standard deviation) maternal age at delivery was 27 ± 5.4 years. About a third of the live births (32%) were to teenage mothers (under age of 18 at delivery). Of the total included 1152 live births, 68 neonates died yielding a NMR of 63 per 1000 live births. The majority of mothers [996 (86%)] were followed by phone call, 112 (10%) were followed by HEW, and the remaining 44 (4%) died while in the study hospitals before discharged. There were 60 dyads lost to follow up, withdrawal, or refusal.

**Table 1:** Sociodemographic characteristics of mothers participated in this study (*N* = 1152)

Characteristics	N (%)	Percent
Hospital		
Ayder Referral	139	12.1
Adwa	198	17.2
Lemlem Karl	147	12.8
Suhl	143	12.4
KahsayAbera	102	8.9
Kidistmaram	213	18.5
Adigrat	210	18.2
Maternal Age at marriage		
< 18	368	31.9
≥ 18	784	68.1
Religion		
Orthodox	1084	94.1
Other	68	5.9
Residence		
Rural	397	34.5
Urban	755	65.5
Educational status		
Unable to read and write	275	23.9
Primary	338	29.3
Secondary	361	31.3
Tertiary	178	15.5
Monthly income		
Poor	327	32.2
Medium	316	31.1
Rich	373	36.7
Marital status		
Married	1071	93.0
Other	81	7.0

Of the total neonates, 121 (10.5%) were low birth weight. Of the neonates who died, 46 (68%) died in health facilities. Of the total neonates, 61 (5.3%) were hypothermic, with 97% of these infants controlled with immediate interventions (see Table 2).

**Table 2:** Pregnancy and neonatal characteristics of neonates delivered in Tigray, April-July, 2014

Characteristics	No	%
Sex		
Male	610	52.9
Female	542	47.1
Distance		
< 5 km	836	76.3
5-0 km	175	16
> =10 km	84	7.7
Place of death		
Health facility	46	67.6
Home	22	32.4
Birth weight		
Low birth weight	121	10.5
Normal	954	82.8
Macrosomia	77	6.7
Current age of mother		
< 20 year	95	8.2
20–24 year	328	28.5
25–35 year	590	57.2
≥ 35 year	139	12.1
GA		
Preterm	93	8.1
Term	990	85.9
Post term	69	6.0
Birth interval		
Not applicable	537	46.6
< Two years	127	11.0
> Two years	488	42.4
Hypothermia		
Yes	61	5.3
No	1091	94.7
Temperature control (n= 61)		
Yes	59	97
No	2	3.0

*GA* gestational age, *km* Kilometer

### Cause of death and cause specific neonatal mortality

We found the leading causes of death to be prematurity [23 (34%)], and asphyxia [21 (31%)] which accounted for 2 of every 3 deaths. The remaining deaths were caused by infections 8 (12%), congenital abnormalities 5 (7%), and other causes 11 (16%). Three-fourths (51) of the total deaths took place during the early neonatal period, primarily due to prematurity and asphyxia. All neonates who died due to congenital abnormalities [5] did so during the early neonatal period. In the late neonatal period, asphyxia, infection, and prematurity were the leading causes of death at 35, 23, and 23% respectively. Though the absolute number of deaths due to infection was the same in both periods (4), the percentage of deaths from this cause was higher in the late (23%) than in the early (8%) neonatal period. Overall, prematurity was the highest reported cause of death in 20 per 1000 live birth whilst congenital abnormality was the least reported cause at 4 per 1000 live births (Table 3).

**Table 3:** Cause-specific numbers of neonatal mortality, percentages and risks in Tigray, Northern Ethiopia 2014

	Early neonatal period		Late neonatal period		Total		
Cause	No. of NM	%	No. of NM	%	No. of NM	%	Risk^a^
Prematurity	19	37	4	23	23	34	20
Asphyxia	15	29	6	35	21	31	18
Infection	4	8	4	23	8	12	7
Congenital	5	10	0.0	0.0	5	7	4
Unknown	8	16	3	18	11	16	9

*LBW* Low birth Weight, *NM* Neonatal mortality

^a^per 1000 live births

### Association of sociodemographic and cause of death for neonatal mortality

There were no significant difference in distribution of deaths between males and female neonates (*P* = 0.943). Infection was higher among newborns from teenage mothers and all infections were occurred among mothers who lived in the rural areas. A substantial number of deaths [13 (43%)] in the urban areas were caused by asphyxia. Nearly half [12 (47%)] of premature infants were found in mothers of the lowest reported monthly income category. On Pearson chi-square analysis of socio-demographic characteristics with cause of death, age at marriage (*P* = 0.004), residence (*P* = 0.05) and distance (*P* = 0.043) were found to be significantly associated (Table 4).

**Table 4:** Association of selected sociodemographic characteristics with cause of death for neonatal mortality

	Prematurity	Asphyxia	Infection	Congenital	Unknown	*P*
Characteristics	NM Freq(%)	NM Freq(%)	NM Freq(%)	NM Freq(%)	NM Freq(%)	
Age						
< 18	2 (9)	6 (27)	6 (27)	2 (9)	6 (27)	0.004
≥ 18	21 (46)	15 (33)	2 (4)	3 (6)	5 (11)	
Sex						
Male	11(32)	12 (35)	4 (12)	2 (6)	5 (15)	0.94
Female	12 (35)	9 (26)	4 (12)	3 (9)	6 (18)	
Residence						
Rural	12 (32)	8 (21)	8 (21)	3 (8)	7 (18)	0.05
Urban	11(37)	13 (43)	0	2 (7)	4 (13)	
Educational status						
Unable to read	7 (27)	5 (19)	6 (23)	2 (8)	6 (23)	0.16
Primary	4 (25)	8 (50)	1 (6)	2 (13)	1 (6)	
Secondary	6 (37)	7 (44)	1 (6)	0	2 (13)	
Tertiary	6 (56)	1 (11)	0	1 (11)	2 (22)	
Income						
Poor	12 (46)	4 (15)	5 (19)	3 (12)	2 (8)	0.09
Medium	3 (15)	8 (40)	1 (5)	2 (10)	6 (30)	
Rich	6 (33)	7 (39)	2 (11)	0	3 (17)	
Marital status						
Married	22 (34)	19 (29)	8(12)	5(8)	11(17)	0.66
Other	1 (33)	2 (67)	0	0	0	
Distance						
< =5 km	6 (20)	11 (38)	4 (14)	4 (14)	4 (14)	0.04
5–10 km	10 (50)	3 (15)	1 (5)	0	6 (30)	
> =10 km	6 (46)	5 (39)	3 (15)	0	0	

*NM* Neonatal mortality, *Freq* frequency

## Discussion

This observational study was conducted in seven randomly selected hospitals of Tigray region, in northern Ethiopia. Both hospital-based clinical diagnosis and verbal autopsies were used to identify the cause of neonatal death. The present study reports that prematurity and asphyxia are the leading causes of early neonatal deaths; whereas, asphyxia, infection, and prematurity are the leading causes of late neonatal deaths.

The cause of deaths identified in our study reaffirms the findings of previous studies from Ethiopia [11, 26] and elsewhere [10, 18, 27, 28]. Despite significant investments [5] made to reduce prematurity caused neonatal mortality, low birth weight, and preterm birth remains leading causes of death. Dealing with prematurity needs highly skilled professionals, and a highly specialized birth environment. Different interventions have proven to decrease deaths related with prematurity and low birth weight such as: management of hypothermia, kangaroo mother care, early breastfeeding, antenatal steroids, and treatment of infections [29, 30]. Hence, there remains an imperative for skills development, equipment, and continuous training of health professionals.. Besides, we speculate that factors such as maternal nutrition [31] and socioeconomics may also contribute to neonatal mortality.

Asphyxia was also a common cause of death both during the early and late neonatal periods, which is consistent with previous studies [4, 9, 17]. Despite the efforts to create a referral linkage between health facilities starting from the health post and home by HEW to specialized hospitals, the main reason for asphyxiated birth in our study area may be related to delayed referral of mothers from lower health care facility and due to lack of prompt resuscitation of neonates. This may indicate lack of competency of HEW and low level health professionals in identifying the risky mothers and providing early referrals. In addition, essential newborn care seemed to be inadequately applied to manage emergency complication of neonates. Strengthening early referrals and improving resuscitation skill of health professionals could reduce neonatal deaths. This study is inconsistent with some studies which identified infection as either the first or second cause of death [32–34] or asphyxia was the leading cause of death [15–17]. The difference may be due to the method of assigning the probable causes of deaths in the current study or smaller sample sizes. Further, some studies may have been conducted in a setting that better managed prematurity and low birth weight.

In our study setting, prematurity and asphyxia were the predominant causes of death in agreement with previous sub-Saharan Africa studies. Overall, this finding suggests a lack of appropriate resuscitation and immediate care of neonates. Similar to a study from India, most neonates from urban area in our study died from asphyxia [35]. The reason could be urban mothers may be less likely to be affected by prematurity due to higher socioeconomic status than their rural counterparts. Hence, this may result in lowering prematurity in the urban areas, and thus a relative increase in asphyxia.

In this study, we documented a relatively low incidence of infections compared to previous studies in Ethiopia and elsewhere [4, 11, 16, 17]. For instance, a recent population based study in rural Tigray region reported infection as the leading cause neonatal death [11]. This discrepancy in major cause of death could be explained by relatively large number of infants in our study population that were born in, or admitted within 6 h, to hospital, where treatment with antibiotics was available.

This study has also identified the cause of death differentials for early and late neonatal mortality. We found that prematurity and low birth weight was the predominant cause for early neonatal mortality. However, infections caused a great proportion of deaths in the late neonatal period. This finding is in accord with a multi-country study of WHO which reported that prematurity and intrapartum complications accounted for most of the early neonatal mortality, while infection and asphyxia caused most of late neonatal mortality [4, 16, 17]. This shows variation of causes of neonatal mortality during early and late neonatal periods. Most deaths were in the first week of life as a result of complication occurring during pregnancy and birth, with many of these deaths being preventable [36]. This may be related to lack of appropriate and immediate management of complications, and lack of skills in resuscitation and care of premature babies. Most infections were among neonates who died at home, which may indicate the absence of appropriate hygiene and lack of postnatal care at home [7].

In contrast to most previous studies, which relied on only the verbal autopsy questionnaires or only the hospital based record, this study used both verbal autopsy and hospital based diagnosis.

However, the study has limitations. One weakness is that the study occurred only in government hospitals, where mothers with complications and neonates with high chance of mortality may have been selectively referred. In the study region, only 27% of mothers deliver in hospitals or another health care facility [7]. Consequently, the pattern of neonatal deaths may be different from community and home deaths which were not be referred; hence, extrapolation of our findings to home and community deaths is significantly limited. In addition, a significant number of deaths with multiple and unidentified causes were categorized as “unknown” for cause of death. Because of the low number of neonatal deaths, and short study duration, it might be difficult to generalize to other similar settings. We had also a 5.2% lost to follow up which might have resulted in an under- or over-estimation of neonatal mortality.

## Conclusion

In the present study, prematurity was the leading cause of neonatal death. Prematurity and asphyxia caused most early neonatal deaths, with asphyxia and infection seen as predominant in late neonatal deaths. However, infections caused relatively fewer neonatal deaths in hospital births. Early age at marriage, distance to care, and residence were associated with cause of deaths. Therefore, improving the obstetric care around birth, improving prompt resuscitation, specialized care, and strengthening the continuum of care could prevent neonatal deaths in the study area.

## Abbreviations

EDHS: Ethiopian health demographic and health survey
ETB: Ethiopian Birr
HEW: Health extension worker
ICD: International classification of disease
NMR: Neonatal mortality rate
WHO: World Health Organization
